# Are adults with chronic pain more likely to develop hypertension than adults without chronic pain? A systematic review and meta-analysis

**DOI:** 10.1016/j.bja.2025.06.006

**Published:** 2025-07-09

**Authors:** Harriet E. Taylor, Joseph C. Salf, Charlotte R. Roper-Marchand, Dhaneesha N.S. Senaratne, Blair H. Smith, Lesley A. Colvin, Jacob George

**Affiliations:** 1 Chronic Pain Research Group, Division of Population Health and Genomics, School of Medicine, University of Dundee, Dundee, UK; 2 NHS Tayside, Ninewells Hospital and Medical School, Dundee, UK; 3 Cardiovascular Research, School of Medicine, University of Dundee, Dundee, UK

**Keywords:** back pain, chronic headache, chronic pain, fibromyalgia, hypertension

## Abstract

**Background:**

An association between persistent pain and blood pressure is increasingly recognised but is not fully understood. In this systematic review, we aimed to establish whether chronic pain (CP) is associated with a diagnosis of hypertension (HTN).

**Methods:**

After prospective registration (PROSPERO ID: CRD42025629486), we searched six databases from inception until January 8, 2025, for studies investigating the association between CP and HTN. Title and abstract screening, full-text review, data extraction, and risk of bias assessment were performed by two independent reviewers. Study quality assessment used the Risk of Bias In Non-randomised Studies of Exposures (ROBINS-E) tool. Meta-analysis was conducted using a random effects model.

**Results:**

From 7973 records, we identified 23 eligible studies for inclusion, of which 20 provided suitable data for meta-analysis (total participants *n*=1 594 264). The pooled odds ratio for HTN in people with CP compared with controls was 1.66 (95% confidence interval [CI] 1.28–2.15) with high inter-study heterogeneity (*I*^2^=99.8%, Cochran *Q*=10 132, *p*<0.001). For specific pain conditions, the pooled odds ratio for HTN was 1.38 (95% CI 1.20–1.58, n = 374 234, heterogeneity: *I*^2^=65.1%, Cochran *Q*=14.34, *p*=0.014) in people with chronic, widespread pain, and 1.56 (95% CI 1.37–1.79, *n*=477 681, heterogeneity: *I*^2^=0%, Cochran *Q*=0.17, *p*=0.92) in people with chronic headache. There was no association between HTN and musculoskeletal pain, lower back pain, or gender.

**Conclusions:**

In line with a growing body of evidence in this field, this systematic review and meta-analysis confirms an association between chest pain and hypertension. Further research is warranted to understand this association and elucidate any mediating factors.

**Systematic review protocol:**

PROSPERO (CRD42025629486).

**Editor’s key points**

• It is widely accepted that acute pain has an immediate effect on blood pressure. However, it is less well understood how persistent pain might impact blood pressure in the long term.
• This analysis adds to a growing body of evidence confirming that chronic pain is associated with a diagnosis of hypertension.
• This has important implications for the management of cardiovascular risk in adults with a diagnosis of chronic pain, but further studies into the mechanisms behind this association are required.

Chronic pain (CP) and hypertension (HTN) are common co-morbidities affecting millions of people worldwide.^1^^,^^2^ CP is defined as pain that persists or recurs for longer than 3 months and is associated with significant emotional distress, functional. disability, or both.^3^^,^^4^ CP can be classified as primary or secondary to underlying conditions. The prevalence of CP varies according to how it is measured, and it varies between countries, with estimates suggesting that CP affects between 10% and 50% of adults globally, with higher rates in old age and in females.^5^, ^6^, ^7^ HTN is defined as a clinic blood pressure (BP) reading of 140/90 mm Hg or higher in combination with either ambulatory BP monitoring (ABPM) or home BP monitoring (HBPM) averages of 135/85 mm Hg or higher.^8^ The association between acute pain and HTN is well established,^9^, ^10^, ^11^ and there is increasing thought to be a related association between CP and HTN.^12^^,^^13^ However, although there are existing review articles investigating the association between CP and multiple long-term conditions^14^^,^^15^ and between CP and cardiovascular disease,^16^^,^^17^ there are no systematic reviews directly interrogating the association between CP and HTN.

Confirming the association between CP and HTN could have significant clinical implications for managing cardiovascular risk in adults with CP. CP has been associated with excess mortality,^18^ and there is a strong association between CP and cardiovascular disease.^16^^,^^17^^,^^19^, ^20^, ^21^, ^22^ Smaller-scale studies have found that there is a positive association between CP and HTN status^23^ and that higher-intensity CP conditions increase the strength of this relationship.^24^ Some studies have suggested that CP may be associated with uncontrolled HTN.^25^ However, these associations have yet to be thoroughly interrogated.

In this systematic review, we aimed to assess whether adults with CP are at greater risk of HTN compared with adults without CP. We have chosen to consider CP as the exposure and HTN as the outcome because of the aforementioned associations of CP with excess mortality, cardiovascular disease, and physiological and psychological morbidities associated with CP. Our secondary outcome was to investigate whether adults with CP are at greater risk of treatment-resistant HTN compared with adults without CP, defined by the International Society of Hypertension and the European Society of Hypertension as uncontrolled BP despite treatment with three agents or controlled BP on four or more agents.^26^

## Methods

### Search strategy and study eligibility

This systematic review and meta-analysis was prospectively registered with the International Prospective Register of Systematic reviews (PROSPERO) on January 8, 2025 (ID: CRD42025629486). We followed the Preferred Reporting Items for Systematic reviews and Meta-Analyses (PRISMA) guidelines. The search was designed to identify studies that examined the risk of HTN in people with CP compared with people without CP. The search strategy was developed with input from subject experts (LC and JG) and an academic librarian, with reference to previously published reviews and the ICD-11 criteria for CP (Supplementary Table 1).^3^^,^^6^^,^^27^ A systematic literature search was performed from database inception until January 8, 2025. The databases searched were PubMed, Cochrane, Embase, PsycINFO, Web of Science, and Scopus.

The search results were imported into Covidence (Veritas Health Innovation, Melbourne, Australia). Duplicates were automatically removed before screening. Two reviewers independently performed title and abstract screening (HT and CR or JS) and full-text review (HT and JS) against the inclusion and exclusion criteria. Disagreements were discussed and resolved with a third reviewer (LC and JG), where necessary. Bibliographies from these studies were also manually screened for additional studies for inclusion.

Studies were included if they were original research involving human participants aged 18 yr or older, investigating the association between CP (exposure) and HTN (outcome). To qualify for inclusion, CP had to be clearly defined as pain lasting longer than 3 months. In addition, HTN had to be defined as a BP of ≥140/90 mm Hg, the current use of antihypertensive medication, or both. We used the UK National Institute for Health and Care Excellence (NICE) clinical BP threshold for diagnosing and treating HTN in adults to define HTN, as an evidence-based and internationally accepted clinical definition of HTN.^8^ We restricted studies to those in the English language. Eligible study designs included randomised controlled trials, cohort studies, case–control studies, cross-sectional studies, and systematic review studies with meta-analysis. Exclusion criteria were case reports, review articles, meeting or conference abstracts, trial protocols, systematic reviews without meta-analysis, non-English language articles, and papers without an available full text. Studies lacking a control group or that did not define HTN or CP as outlined above were also excluded. We excluded studies where HTN was a self-reported diagnosis.^28^

### Data analysis

Data were extracted independently by two reviewers (HT and JS) using a predefined tool into Microsoft Excel (Microsoft Corporation, Redmond, WA, USA). Discrepancies were resolved by discussion with a third reviewer (LC or JG). Data extraction included the study details (author, year, study design, country, and sample size), sample characteristics (average age and % female), CP details (type, definition, severity, impact, and treatment including analgesic use), and HTN details (definition, severity, and treatment). The exposure was CP, and the outcome was HTN. For meta-analysis, extraction of the number of exposed, the number of controls, the number of exposed with HTN, the number of controls with HTN, the odds ratio (OR), the 95% confidence interval (CI), and the *P*-value was completed. Where an adjusted OR was provided, this was used for analysis. The exception to this was when the author adjusted for mediating factors (e.g. analgesic use); in these cases, the unadjusted OR was used. The corresponding authors were contacted directly to request any relevant missing data, where necessary.

Quality assessment and risk of bias were assessed using the Risk of Bias In Non-randomised Studies of Exposures (ROBINS-E) tool, adapted to the relevant study type, as per Cochrane guidance.^29^^,^^30^ The risk of bias for each study was assessed across seven domains (confounding, measurement of exposure, participant selection, post-exposure interventions, missing data, measurement of outcome, and selection of the reported result), and the overall risk of bias was determined after the ROBINS-E algorithm. This was done independently by two reviewers (HT and JS), and any discrepancies were settled by consensus discussion. For this review, it was determined that it was necessary to have adjusted for at least age and sex. Studies that did not adjust for these would be considered ‘high’ risk in the confounding domain. A directed acyclic graph (DAG) showing our assumptions is included in Supplementary Figure 1.^31^

All statistical analyses were performed using R version 4.2.3 for Windows using the RStudio integrated development environment (version 2024.12.1-563, RStudio Team, Boston, MA, USA). To avoid duplication of participant data where studies analysed the same population sample, we selected the study with the most complete reporting of raw data for meta-analysis. Meta-analysis of the ORs was performed using the meta package, and the random-effects model was used to calculate the pooled risk.^32^ We conducted an overall comparison across all included papers; where there were mixed CP cohorts, the group with the largest sample population was chosen for meta-analysis. We also made comparisons according to pain subtype, including chronic widespread pain (CWP), chronic headache, burning mouth syndrome (BMS), and chronic musculoskeletal (MSK) pain. Studies including adults with fibromyalgia syndrome (FMS) were included in the CWP analysis.^33^^,^^34^ For the chronic MSK pain analysis, we combined results from studies that examined chronic MSK pain, chronic back pain (CBP), chronic joint pain, and chronic knee pain (CKP). We also performed a subgroup analysis by gender. Study heterogeneity was calculated using the *I*^2^ statistic, the Cochran *Q* statistic, and the Cochran *Q P*-value.

## Results

**Fig 1:**
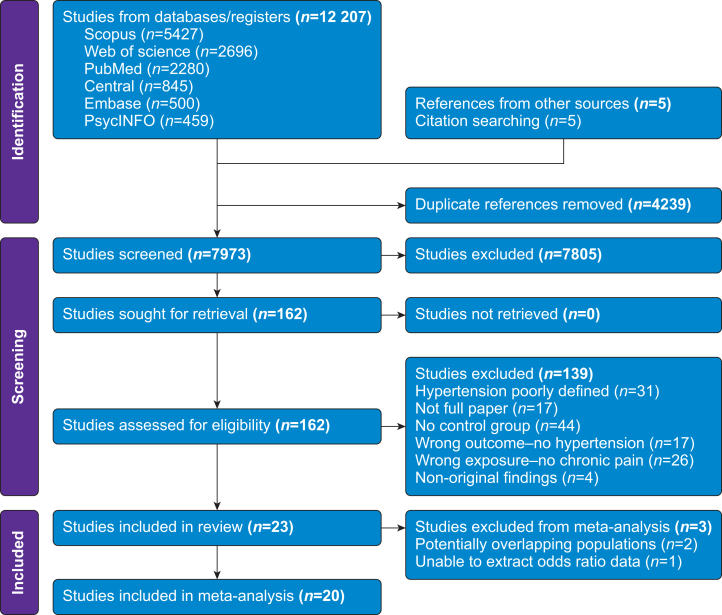
Flow chart of study selection for systematic review and meta-analysis.

The search generated 12 212 studies, of which 4239 were duplicates. After abstract screening, 162 full-text articles were compared in detail against the eligibility criteria, and 23 met the inclusion criteria for the systematic review (Fig. 1). The studies were all observational in design, including cohort (*n*=9), cross-sectional (*n*=8), and case–control (*n*=6) studies. Nine of the included studies were cohort studies; however, only cross-sectional data on HTN were available to extract, and so temporal data could not be analysed. The total number of participants, excluding studies with overlapping cohorts, was 1 693 073. The study characteristics are presented in Supplementary Table 2. The study included European (*n*=13), Asian (*n*=8), South American (*n*=1), and North American (*n*=1) populations. Most studies had predominantly female populations (median 58.6% female, interquartile range [IQR] 53.3–82.9%). For the meta-analysis, three studies were excluded after contacting relevant authors for clarification, with no response received, because of concerns regarding overlapping sample populations^35^^,^^36^ and insufficient data for meta-analysis.^37^

### Risk of bias assessment

Risk of bias assessment was conducted using the ROBINS-E tool (Supplementary Table 3). Overall, four studies were considered ‘low’ risk,^38^, ^39^, ^40^, ^41^ 13 studies had ‘some concerns’,^25^^,^^35^^,^^37^^,^^42^, ^43^, ^44^, ^45^, ^46^, ^47^, ^48^, ^49^, ^50^, ^51^ and six studies were considered ‘high’ risk.^36^^,^^52^, ^53^, ^54^, ^55^, ^56^ The key area of concern was in domain 1 (risk of bias attributable to confounding), with studies considered ‘high’ risk if they did not control for both age and sex,^36^^,^^52^^,^^53^^,^^55^^,^^56^ or by controlling for mediators such as analgesic use.^54^ One study scored ‘high’ in domain 3 (risk of bias attributable to selection of participants) as it recruited participants from a very narrow population (dialysis-dependent patients).^53^

### Exposure: chronic pain

The studies included a wide variety of CP diagnoses: FMS (*n*=4),^38^^,^^40^^,^^43^^,^^55^ CWP (*n*=1),^48^ chronic headache (*n*=2),^25^^,^^39^ CBP (*n*=4),^36^^,^^41^^,^^46^^,^^53^ chronic MSK pain (*n*=2),^37^^,^^54^ BMS (*n*=3),^35^^,^^42^^,^^56^ bladder pain syndrome (BPS, *n*=1),^44^ and mixed CP cohorts (*n*=6).^45^^,^^47^^,^^49^, ^50^, ^51^, ^52^ Full details of the CP diagnostic criteria for each study are found in Supplementary Table 4. In the included studies, the prevalence of CP was 21.2% (*n*=346 027). In the CP group, the overall prevalence of HTN was 36.8% (*n*=116 641), whereas in the non-CP group, it was 19.8% (*n*=142 017).

### Primary outcome: all chronic pain types and hypertension meta-analysis

**Fig 2:**
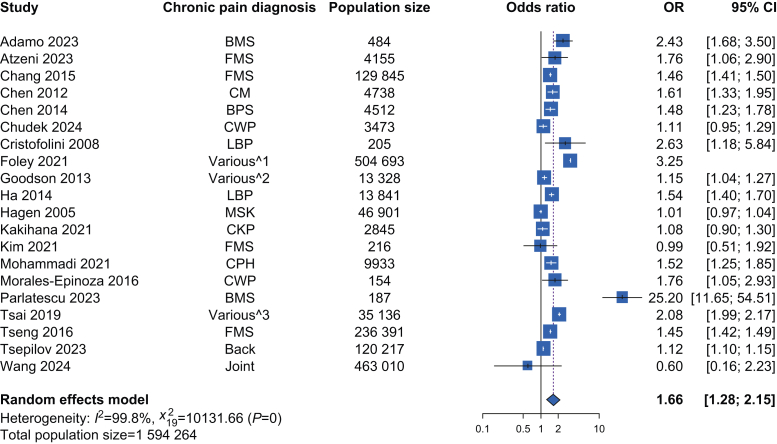
Meta-analysis of the risk of hypertension in adults with chronic pain compared with adults without chronic pain. BMS, burning mouth syndrome; BPS, bladder pain syndrome; CI, confidence interval; CKP, chronic knee pain; CM, chronic migraine; CPH; chronic primary headache; CWP, chronic widespread pain; FMS, fibromyalgia syndrome; HTN, hypertension; LBP, lower back pain; MSK, musculoskeletal; OR, odds ratio. 1 = arthritis, back/neck, headache, trauma, neuropathic, and bone. 2 = head, neck, back, chest, abdomen, limbs, and other sites of pain not specified. 3 = osteoarthritis, spinal disorders, peripheral vascular disease, osteoporosis, gout, headache, diabetic neuropathy, malignancy, rheumatoid arthritis, and pressure ulcers.

Twenty studies (involving 1,594,264 participants) were included in the meta-analysis exploring associations between HTN in people with CP compared with people without CP (Table 1), with a pooled OR of 1.66 (95% CI 1.28–2.15) (Fig. 2) for HTN in people with CP compared with people without CP.^25^^,^^38^, ^39^, ^40^, ^41^, ^42^, ^43^, ^44^, ^45^, ^46^, ^47^, ^48^, ^49^, ^50^, ^51^, ^52^, ^53^, ^54^, ^55^, ^56^ Heterogeneity was very high among the included studies in the overall meta-analysis (*I*^2^=99.8%, Cochran *Q*=10 132, *P*<0.001). The results were not substantially altered after the exclusion of studies with high risk of bias (OR 1.58, 95% CI 1.34–1.87, *n*=1 543 282, heterogeneity: *I*^2^=99.8%, Cochran *Q*=8,616, *P*<0.001).^51^, ^52^, ^53^, ^54^^,^^56^ To further assess this heterogeneity, we used a L'Abbé plot analysis (Supplementary Fig. 2), which identified two potential outliers.^53^^,^^56^ We then investigated the presence of publication bias and other reporting biases visually using a Funnel plot (Supplementary Fig. 3). This demonstrated asymmetry; however, the results of Egger’s test (t=–1.14, df=18, *P*=0.27) suggested that this was not statistically significant.^57^

**Table 1:** Studies identified in the systematic review, reporting risk of hypertension in adults with chronic pain compared with adults without chronic pain. BMS, burning mouth syndrome; BPS/IC, bladder pain syndrome/interstitial cystitis; CBP, chronic back pain; CI, confidence interval; CP, chronic pain; CWP, chronic widespread pain; DBP, diastolic blood pressure; FMS, fibromyalgia syndrome; HTN, hypertension; OR, odds ratio; SBP, systolic blood pressure. ∗Values calculated from study data.

Author and year	Exposure (CP)	Sample population	Sample size; *n* included	Risk of outcome (HTN) as OR	95% CI
Adamo^42^ 2023	BMS	242 adults with BMS presenting to a dental clinic and 242 age- and sex-matched controls	500; 484	2.43∗ (unadjusted)	1.67–3.50∗
Atzeni^38^ 2023	FMS	62 female adult outpatients with FMS and 4093 age-matched female controls from a representative Italian population sample	4155; 4155	1.76∗ (unadjusted)	1.06–2.90∗
Canfora^35^ 2022	BMS	250 female adults with BMS recruited by an oral medicine department and 250 age-matched female controls	535; 500	2.40∗ (unadjusted)	1.67–3.46∗
Chang^43^ 2015	FMS	25 969 adults with FMS and no psychiatric history and 103 876 age- and sex-matched controls (from the Taiwan National Health Insurance Research Database); Study 1 data used	129 845; 129 845	1.46∗ (unadjusted)	1.41–1.50∗
Chen^39^ 2012	Chronic migraine	948 adults with chronic migraine and 3790 age-, sex-, and income-matched controls (from the Taiwan National Health Insurance Research Database)	4891; 4738	1.61∗ (unadjusted)	1.33–1.95∗
Chen^44^ 2014	BPS/IC	752 female adults with BPS/IC and 3760 age-matched female controls (from the Taiwan Longitudinal Health Insurance Database 2000)	1 000 000; 4512	1.48∗ (unadjusted)	1.23–1.78∗
Chudek^52^ 2024	CWP and chronic regional pain	Elderly Polish population sample (from a national multicentre study on ageing)	4979; 3473	1.11∗ (unadjusted)	0.95–1.29∗
Cristofolini^53^ 2008	Chronic lower back pain	Adults with end-stage renal disease receiving haemodialysis three times per week (recruited from dialysis unit)	234; 205	2.63∗ (unadjusted)	1.18–5.84∗
Foley^51^ 2021	Various—musculoskeletal/arthritis, back/neck, headache, musculoskeletal trauma, neuropathic, bone, and other	Canadian residents eligible for Medical Care Plan benefits (health administrative data)	516 729; 504 693	3.25 (adjusted: sex, regional health authority, and rural/urban residential location)	3.21–3.30
Goodson^45^ 2013	Various —head, neck, back, chest, abdomen, limbs, and other sites of pain not specified	Volunteer-based family-structured population sample (Generation Scotland: The Scottish Family Health study)	23 960; 13 328	DBP: 1.15 (adjusted: age and gender)	1.04–1.26
Ha^46^ 2014	Chronic lower back pain	Nationally representative population sample of South Koreans aged 20–89	13 841; 13 841	1.54∗ (unadjusted)	1.40–1.70∗
Hagen^54^ 2005	Chronic musculoskeletal pain	General population sample (two consecutive public health studies: HUNT-1 and HUNT-2); HUNT-2 crude data for SBP ≥140 used for this analysis	47 556; 46 901	1.01∗ (unadjusted)	0.97–1.04∗
Heuch^36^ 2014	Chronic lower back pain	General population sample (from health surveys: HUNT-2 cross-sectional and HUNT-3 prospective); cross-sectional data from HUNT-2 used for this analysis	39 872	SBP: 0.97∗ (unadjusted)	0.92–1.02∗
Kakihana^47^ 2021	Chronic knee pain and chronic lower back pain	General population sample (circulatory risk study)	2970; 2845	Chronic knee pain: 1.08 (adjusted: age, sex, area, overweight, inactivity, smoking status, drinking status, mental stress, depressive status, and job)	0.89–1.29
Kim^55^ 2021	FMS	58 female adults with FMS (from a rheumatology outpatient clinic) and 158 healthy controls (from an annual health check-up service)	216; 216	0.99∗ (unadjusted)	0.51–1.92∗
Meert^37^ 2025	Chronic musculoskeletal pain	General population sample (from a longitudinal cohort study)	4519; 3437	0.94 (adjusted: sex and age)	0.84–1.06
Mohammadi^25^ 2021	Chronic primary headache	General population sample (part of a national prospective epidemiological study)	10 000; 9932	1.52 (adjusted: age, gender, education, occupation, smoking, alcohol, opioids, BMI, physical activity, cholesterol, diabetes mellitus, triglycerides, and FHx chronic headache)	1.25–1.85
Morales-Espinoza^48^ 2016	CWP	130 adults with CWP and 124 age- and sex-matched controls; selected from adults attending three urban primary care centres in Spain	16 229; 154	1.76∗ (unadjusted)	1.05–2.93
Parlatescu^56^ 2023	BMS	99 adults with BMS and 88 controls aged ≥50 yr recruited from a dentistry faculty in Romania	187; 187	25.2∗ (unadjusted)	11.6–54.5∗
Tsai^50^ 2019	Various—osteoarthritis (24.2%), spinal disorders (22.4%), peripheral vascular disease (14%), osteoporosis (9.5%), gout, malignancy, headache, diabetic neuropathy, rheumatoid arthritis, and pressure ulcer (classified according to ICD-9-CM codes)	17 568 adults ≥65 yr with chronic pain and 17 568 age and sex-matched controls (Longitudinal Health Insurance Database 2000, a subset of the Taiwan National Health Insurance Research Database)	2 000 000; 35 136	2.08∗ (unadjusted)	1.99–2.17∗
Tseng^40^ 2016	FMS	47 279 adults with newly diagnosed FMS between 2000 and 2002 and 189 112 age- and sex-matched controls (Longitudinal Health Insurance Database 2000, a subset of the Taiwan National Health Insurance Research Database)	1 000 000; 236 391	1.45∗ (unadjusted)	1.42–1.49∗
Tsepilov^41^ 2023	CBP	21 543 adults with CBP and 98 647 controls (from UK Biobank)	120 217; 120 217	1.12 (adjusted: sex, age, and genotyping batch)	1.10–1.15∗
Wang^49^ 2024	Various—chronic headache, chest pain, abdominal pain, joint pain, back pain, limb pain, and multisite chronic pain	Two-sample Mendelian Randomisation study using GWAS data from UK BioBank	1 873 960; 463 010 (joint)	Joint: 0.599 (unadjusted)	0.16–2.23

### Primary outcome: different types of chronic pain and hypertension meta-analysis

**Fig 3:**
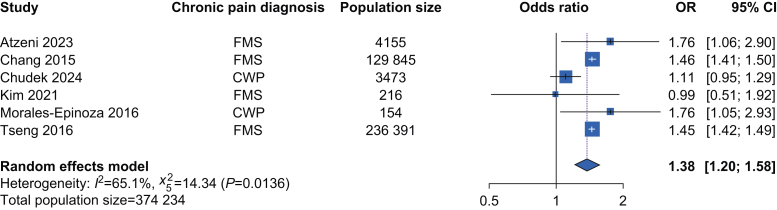
Meta-analysis of the risk of hypertension in adults diagnosed with chronic widespread pain compared with adults without chronic widespread pain. CI, confidence interval; CWP, chronic widespread pain; FMS, fibromyalgia syndrome; HTN, hypertension; OR, odds ratio.

**Fig 4:**
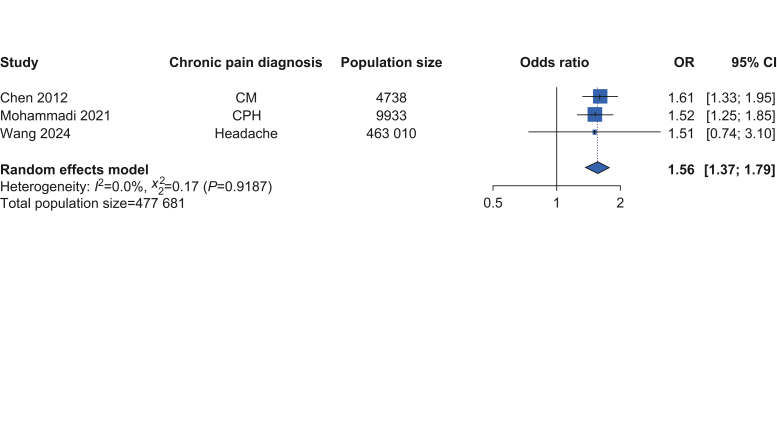
Meta-analysis of the risk of hypertension in adults with chronic headache compared with adults without chronic headache. CI, confidence interval; CM, chronic migraine; CPH, chronic primary headache; HTN, hypertension; OR, odds ratio.

Subgroup analysis found that in adults with CWP (*n*=374 234), the pooled OR for HTN compared with adults without CP was 1.38 (95% CI 1.20–1.58) (Fig. 3). In adults with chronic headache (*n*=477 681), the pooled OR for HTN was 1.56 (95% CI 1.37–1.79) (Fig. 4). There was no significantly altered risk of HTN in adults with CBP (OR 1.27, 95% CI 0.96–1.68, *n*=251 667), combined chronic MSK pain (OR 1.19, 95% CI 0.98–1.44, *n*=647 019), or BMS (OR 7.62, 95% CI 0.77–75.51, *n*=671). No significant OR for HTN was identified in the gender sub-group analysis. Six studies included data for females (*n*=37 389), with three studies having exclusively female participants^38^^,^^44^^,^^55^ and three studies separating the sexes.^42^^,^^46^^,^^54^ This gave a pooled OR for HTN in females with CP of 1.17 (95% CI 0.96–1.43) compared with females without CP. Three studies included separate data for males (*n*=27 820),^42^^,^^46^^,^^54^ with a pooled OR for HTN in males with CP of 0.83 (95% CI 0.61–1.11) compared with males without CP.

### Primary outcome: severity of chronic pain and hypertension

Three studies analysed the association between the severity of CP and the risk of HTN. Because of heterogeneous data collection methods and potential population overlap, meta-analysis could not be conducted for this association. In two studies examining BMS, there was no significant difference in the numeric rating scale (NRS) or short-form McGill pain questionnaire between people with BMS with and without HTN.^35^^,^^42^ Another study found that there was a 14% greater risk of raised diastolic BP (DBP) >90 mm Hg in those reporting high-intensity CP compared with those reporting low-intensity CP.^45^ The association between CP impact and HTN was assessed in two studies; there were no significant differences in HTN prevalence associated with pain-related anxiety, depression, or sleep scores in adults with BMS compared with adults without BMS.^35^^,^^42^

### Secondary outcome: chronic pain and treatment-resistant hypertension

The included studies contained sparse and heterogeneous data on treatment-resistant HTN. Therefore, meta-analysis of our secondary outcome could not be completed. Four studies analysed antihypertensive regimens specifically. In two of these, people with BMS were found to be more frequent users of angiotensin receptor blockers (15.3% *vs* 7.4%, *P*=0.0009)^42^ and ACE inhibitors (16.4% *vs* 8.4%, *P*=0.009)^35^ compared with people without BMS. They also found an association with BMS and the use of a single antihypertensive agent (30% *vs* 19.2%, *P*=0.007), but this was lost when two or more antihypertensive agents were used.^35^ Conversely, one study of people with chronic primary headache found no significant association between this and the use of any type of antihypertensive medication.^25^

Four of the included studies investigated whether the severity of HTN was associated with CP. One study demonstrated a significantly lower prevalence of chronic MSK pain in all age groups in those with systolic BP (SBP) ≥150 mm Hg^54^ and found that the risk of chronic MSK pain decreased linearly with increasing BP.^54^ Similarly, a further study found that the risk of chronic LBP was significantly lower among people with HTN with SBP ≥160 mm Hg or DBP ≥100 mm Hg compared with people without HTN (OR 0.7, 95% CI 0.49–0.99); participants with SBP 140–159 or DBP 90–99 did not have a significantly lower risk of chronic LBP compared with people without HTN.^47^ One study investigated whether the duration (>6 yr) and control of HTN affected the risk of chronic primary headache.^25^ They found that people with chronic primary headache, and not those with episodic headache, were at higher risk of both long-duration uncontrolled HTN (OR 1.47, 95% CI 1.03–2.09) and long-duration controlled HTN (OR 1.47, 95% CI 1.16–2.39). Finally, the association between the grade of HTN and CP was investigated in one study.^56^ This found a lower proportion of people with BMS and HTN had grade 3 HTN (SBP >180 mm Hg or DBP >110 mm Hg) compared with hypertensive controls without BMS (10.7% *vs* 37.5%).

## Discussion

### Chronic pain and hypertension

This systematic review and meta-analysis has demonstrated a significant positive association between CP and HTN across a large sample population with a large range of CP conditions. Overall, we found 66% higher odds of HTN in people with CP compared with people without CP. These findings support a growing body of evidence linking CP and HTN. For example, three previous meta-analyses have found a significant association between CP and cardiovascular disease risk.^16^^,^^20^^,^^22^ In addition, several population-based studies have found that HTN is a mediating factor in the relationship between CP and cardiovascular morbidity.^58^, ^59^, ^60^, ^61^ In our analysis, the association between CP and HTN was evident in people with CWP (across six included studies) and chronic headache (across three included studies). This is in line with previous research where FMS has been associated with increased cardiovascular mortality in a meta-analysis.^62^ In addition, chronic headache has been associated with increased prevalence of HTN.^63^ Notably, the OR for CP overall was higher than for both CWP and chronic headache. This could be because in the overall analysis, we have a broader sample population and therefore capture a larger spectrum of CP-related cardiovascular effects. In addition, there could be a mechanistic component, with the CWP and chronic headache analyses potentially not accounting for physiological processes (e.g. injury and inflammation) associated with other forms of CP, which could predispose to HTN.^64^ Another contributing factor could be that chronic headache may be a symptom of uncontrolled HTN, introducing a bidirectional causality that may influence the overall OR.^65^

When assessing CBP and BMS individually, we found no significant association with HTN. However, some studies suggest that in these conditions, greater pain severity is associated with a higher resting BP.^66^^,^^67^ On combining CBP, joint pain, and knee pain across six included studies, we found no association between chronic MSK pain and HTN. This is on a background of conflicting evidence, with a previous meta-analysis suggesting chronic MSK pain increases the risk of cardiovascular disease.^20^ Our analysis found no significantly greater risk of HTN associated with adults with CP of either gender, when analysed separately. This was from a small subset of studies; three studies had gender-differentiated results, and three looked at female data alone. The literature on this is sparse, but it has been suggested that, in acute pain, females exhibit greater HTN-associated hypoalgesia than males,^68^ warranting further investigation of this in CP.

Although still a matter of debate, there are thought to be pathophysiological mechanisms that underlie this association between CP and HTN, the most well-documented being the dysregulation of the baroreflex response (Supplementary Fig. 4). In acute pain, nociceptive stimuli activate the sympathetic nervous system (SNS) and the hypothalamic–pituitary–adrenal axis, leading to a cardiovascular and adrenergic response, which causes a transient increase in BP. In healthy individuals, arterial baroreceptors detect these increases in systemic BP and, via the nucleus of the solitary tract, inhibit SNS activity and reduce pain perception via stimulation of the inhibitory descending pain pathways.^10^ This results in an inverse linear relationship between BP and acute pain perception, known as HTN-associated hypoalgesia.^10^^,^^69^^,^^70^ This relationship is not maintained in CP, where there are known changes in the balance between descending inhibition and facilitation in nociceptive pathways.^17^^,^^71^, ^72^, ^73^ In CP, there can be SNS overactivation from both direct spinal nociceptive inputs and chronic emotional stress.^74^^,^^75^ Rather than HTN-associated hypoalgesia, in CP, it has been shown that higher BP at rest is associated with greater sensitivity to acute pain and a higher intensity of CP.^76^ It is hypothesised that the physiological response to CP, involving prolonged compensatory changes in cardiovascular, endocrine, autonomic, immune, and somatosensory systems, eventually progresses to maladaptive changes in pain inhibition and BP.^10^^,^^77^^,^^78^ For example, it has been suggested that there is impaired baroreflex sensitivity^12^^,^^13^ and impaired descending inhibitory pain pathways^71^ in CP, potentially resulting in a maladaptive feedback loop that sustains both pain and HTN.

In addition to altered physiology in CP syndromes, lifestyle factors (e.g. sleep quality, physical activity, substance use, psychiatric conditions, and diet) and medication use may also alter the risk of HTN. The mediating role of lifestyle factors is supported by a prospective population-based study, which found that the higher cardiovascular mortality risk associated with CP became insignificant after adjusting for lifestyle factors.^79^ Medications used in the treatment of both CP and its common comorbid conditions have been shown to have effects on BP. In particular, there is an association between HTN and some commonly used analgesics (e.g. non-steroidal anti-inflammatory drugs and gabapentinoids)^80^ and some antidepressants (e.g. tricyclic antidepressants and serotonin–norepinephrine reuptake inhibitors)^81^ via enhanced adrenergic transmission.^82^ Details of analgesic use were collected as part of our data extraction process. However, there were insufficient data to analyse the association between analgesic use and HTN diagnoses in adults with CP. Moreover, illicit drugs such as cocaine can also affect BP, and several studies have found increased rates of cocaine use in adults with CP compared with adults without CP.^83^^,^^84^ In addition, common comorbidities in adults with CP (e.g. sleep apnoea) have also been associated with HTN.^85^ Studies included in our analysis did not have sufficient data on medication use and comorbidities in the context of the risk of HTN to delineate such associations. However, we recognise their potential direct mechanistic contribution to HTN in people with CP.

### Chronic pain and treatment-resistant hypertension

Although in this analysis, the exposure was CP and the outcome was HTN, our review identified an element of bidirectionality in this association. Five studies found that HTN, when prolonged or severe, may be protective against CP.^25^^,^^35^^,^^47^^,^^54^^,^^56^ One population-based study contradicts this hypothesis, finding that males with uncontrolled HTN were at higher risk of musculoskeletal complaints.^86^ Conversely, one experimental study using a monoarthritic rat model of CP found that hypertensive animals had lower levels of inflammation and lower pain sensitivity compared with normotensives.^87^ It should be noted that all hypertensive rat models in this study used artificial modification of the renin–angiotensin–aldosterone system (RAAS). This is a limitation, as the RAAS is also involved in pain perception; it has been found that angiotensin II precipitates neuropathic pain as well as increasing BP^88^ and that angiotensin-converting enzyme (ACE) inhibitors and angiotensin receptor blockers (ARBs) can reduce pain sensitivity.^89^ Interestingly, two of the included studies in our review found that people with CP used ARBs and ACE inhibitors more frequently than controls.^35^^,^^42^

### Strengths and limitations

A major strength of this analysis is that there were sufficient studies with adequate homogeneity to conduct a meta-analysis. The analysis also incorporated data from a large sample of people with CP. In addition, most studies (*n*=14) used population-based samples. However, only three studies were from low-income countries, 13 studies were from European cohorts, and four studies included only female participants, reducing the generalisability of our findings. This meta-analysis has some important limitations that must be considered when interpreting the results. For example, no inferences about causal relationships can be made from this analysis because of the inclusion of exclusively observational data. In addition, there is significant inter-study heterogeneity, likely attributed to the variability in sample sizes, study designs (cross-sectional, cohort, and case–control), and the various definitions for CP included. Our methodology attempted to reduce heterogeneity by maintaining strict definitions for CP and HTN. However, the nature of our analysis meant some heterogeneity could not be avoided through the inclusion of a variety of CP subtypes. There were also significant discrepancies between studies in how they controlled for covariates, which may have introduced confounding bias to the analysis. Another limitation is that analgesic use could not be assessed as a mediating factor because none of the included studies had data on analgesics in the context of HTN in adults with CP. In addition, our resources and timing precluded consideration of studies that were not reported in the English language, and we are unable to assess the extent of bias that this will have created.

### Conclusions and future studies

In summary, this systematic review and meta-analysis supports an association between CP and HTN across a large sample population and range of CP diagnoses. This association was particularly evident in chronic headache and CWP. However, we were unable to consolidate evidence linking treatment-resistant HTN and CP, and there was significant heterogeneity in our results. Nevertheless, this finding supports an ever-growing body of evidence in this field and highlights the need to further elucidate the association between CP and HTN. Given the high prevalence of CP and HTN globally, these results highlight a potentially significant public health risk, and appreciation of this will help inform comprehensive management strategies in this patient group. Regarding future research, prospective population-based studies investigating the causal relationship between CP and HTN are now required. Ideally, these would control for all important confounding variables to explore the causality of this association. There is also a need for studies investigating the association between CP and treatment-resistant HTN, the reverse association between HTN and CP, and whether therapies for CP could ameliorate HTN risk or vice versa.

## Authors’ contributions

Study conception and design: HET, JG, LAC, BHS

Acquisition of data: HET, JCS, CRR

Analysis and interpretation of data: HET, JCS, DNS, JG, LAC

Drafting, revision, and final approval of the manuscript: HET, DNS, LAC, JG, BHS

## Funding

Multimorbidity Doctoral Training Programme for Health Professionals, Wellcome Trust (223499/Z/21/Z to DS). Advanced Pain Discovery Platform (APDP), UK Research and Innovation (UKRI); Versus Arthritis; Eli Lilly, and APDP grant, [Partnership for Assessment and Investigation of Neuropathic Pain: Studies Tracking Outcomes, Risks and Mechanisms (PAINSTORM) consortium (MR/W002388/1)] (to BS and LC). APDP grant[Consortium Against Pain Inequality (MR/W002566/1)] (to LC).

## Declarations of interest

LAC is a member of the *BJA Open* editorial board. The other authors declare that they have no competing interests.
