# Supplementary material for: Are adults with chronic pain more likely to develop hypertension than adults without chronic pain? A systematic review and meta-analysis
Source: Br J Anaesth. 2025 Jul 9;135(3):685–96. doi: 10.1016/j.bja.2025.06.006 (PMC12489386; doi:10.1016/j.bja.2025.06.006)
Supplement: Multimedia component 1 [file mmc1.docx]

**Are adults with chronic pain more likely to develop hypertension when compared to adults without chronic pain? A systematic review and meta-analysis**

Harriet E Taylor, Joseph C Salf, Charlotte R Roper-Marchand, Dhaneesha NS Senaratne, Blair H Smith, Lesley A Colvin, Jacob George

Supplementary material: Table of Contents

Table S1: Search Strategy2

Table S2: Study characteristics7

Table S3: Risk of bias assessment (ROBINS-E)………………………………………………………………………………………………………………… 10

Table S4: Study details of exposure and outcome and associated odds ratios…...……………………………………………………………………………… 11

Figure S1: Directed Acyclic Graph (DAG)………………………………………………………………………………………………………….………….. 18

Figure S2: L’Abbé plot comparing CP and non-CP event rates (HTN) across studies…………………………………………………………………….......... 19

Figure S3: Funnel plot of study Odds Ratio against the Standard Error………………………………………………………………………………………… 20

Figure S4: Conceptual diagram illustrating the pathways involved in HTN-associated hypoalgesia…………………………………………………………... 21

References………………………………………………………………………………………………………………………………………………………. 22

# **Table S1. Search Strategy**

The search term was developed in discussion with co-author L.C. and the University of Dundee Academic Librarian (S.M.). The search was modified for each database searched using the Polygot Search Translator ^1^.

| **No.** | **PubMed** | **Cochrane** | **Embase** | **PsycINFO** | **Web of Science** | **Scopus** |
| --- | --- | --- | --- | --- | --- | --- |
| 1 | complex regional pain syndrome[Title/Abstract] | complex regional pain syndrome:ti,ab | complex regional pain syndrome:ti,ab | complex regional pain syndrome:ti,ab | complex regional pain syndrome:ti,ab | complex regional pain syndrome:ti,ab |
| 2 | burning mouth syndrome[Title/Abstract] | burning mouth syndrome:ti,ab | burning mouth syndrome:ti,ab | burning mouth syndrome:ti,ab | burning mouth syndrome:ti,ab | burning mouth syndrome:ti,ab |
| 3 | chronic migraine[Title/Abstract] | chronic migraine:ti,ab | chronic migraine:ti,ab | chronic migraine:ti,ab | chronic migraine:ti,ab | chronic migraine:ti,ab |
| 4 | fibromyalg*[Title/Abstract] | fibromyalg*:ti,ab | fibromyalg*:ti,ab | fibromyalg*:ti,ab | fibromyalg*:ti,ab | fibromyalg*:ti,ab |
| 5 | chronic widespread pain[Title/Abstract] | chronic widespread pain:ti,ab | chronic widespread pain:ti,ab | chronic widespread pain:ti,ab | chronic widespread pain:ti,ab | chronic widespread pain:ti,ab |
| 6 | functional GI[Title/Abstract] | functional GI:ti,ab | functional GI:ti,ab | functional GI:ti,ab | functional GI:ti,ab | functional GI:ti,ab |
| 7 | functional abdominal pain[Title/Abstract] | functional abdominal pain:ti,ab | functional abdominal pain:ti,ab | functional abdominal pain:ti,ab | functional abdominal pain:ti,ab | functional abdominal pain:ti,ab |
| 8 | IBS[Title/Abstract] | IBS:ti,ab | IBS:ti,ab | IBS:ti,ab | IBS:ti,ab | IBS:ti,ab |
| 9 | irritable bowel syndrome[Title/Abstract] | irritable bowel syndrome:ti,ab | irritable bowel syndrome:ti,ab | irritable bowel syndrome:ti,ab | irritable bowel syndrome:ti,ab | irritable bowel syndrome:ti,ab |
| 10 | vulvodynia[Title/Abstract] | vulvodynia:ti,ab | vulvodynia:ti,ab | vulvodynia:ti,ab | vulvodynia:ti,ab | vulvodynia:ti,ab |
| 11 | chronic neuropathic pain[Title/Abstract] | chronic neuropathic pain:ti,ab | chronic neuropathic pain:ti,ab | chronic neuropathic pain:ti,ab | chronic neuropathic pain:ti,ab | chronic neuropathic pain:ti,ab |
| 12 | chronic postoperative pain[Title/Abstract] | chronic postoperative pain:ti,ab | chronic postoperative pain:ti,ab | chronic postoperative pain:ti,ab | chronic postoperative pain:ti,ab | chronic postoperative pain:ti,ab |
| 13 | chronic post-surgical pain[Title/Abstract] | chronic post-surgical pain:ti,ab | chronic post-surgical pain:ti,ab | chronic post-surgical pain:ti,ab | chronic post-surgical pain:ti,ab | chronic post-surgical pain:ti,ab |
| 14 | chronic postsurgical pain[Title/Abstract] | chronic postsurgical pain:ti,ab | chronic postsurgical pain:ti,ab | chronic postsurgical pain:ti,ab | chronic postsurgical pain:ti,ab | chronic postsurgical pain:ti,ab |
| 15 | chronic cancer pain[Title/Abstract] | chronic cancer pain:ti,ab | chronic cancer pain:ti,ab | chronic cancer pain:ti,ab | chronic cancer pain:ti,ab | chronic cancer pain:ti,ab |
| 16 | cancer pain[Title/Abstract] | cancer pain:ti,ab | cancer pain:ti,ab | cancer pain:ti,ab | cancer pain:ti,ab | cancer pain:ti,ab |
| 17 | chronic TMD[Title/Abstract] | chronic TMD:ti,ab | chronic TMD:ti,ab | chronic TMD:ti,ab | chronic TMD:ti,ab | chronic TMD:ti,ab |
| 18 | chronic temporomandibular[Title/Abstract] | chronic temporomandibular:ti,ab | chronic temporomandibular:ti,ab | chronic temporomandibular:ti,ab | chronic temporomandibular:ti,ab | chronic temporomandibular:ti,ab |
| 19 | chronic orofacial pain[Title/Abstract] | chronic orofacial pain:ti,ab | chronic orofacial pain:ti,ab | chronic orofacial pain:ti,ab | chronic orofacial pain:ti,ab | chronic orofacial pain:ti,ab |
| 20 | Chronic primary pain[Title/Abstract] | Chronic primary pain:ti,ab | Chronic primary pain:ti,ab | Chronic primary pain:ti,ab | Chronic primary pain:ti,ab | Chronic primary pain:ti,ab |
| 21 | chronic pain[Title/Abstract] | chronic pain:ti,ab | chronic pain:ti,ab | chronic pain:ti,ab | chronic pain:ti,ab | chronic pain:ti,ab |
| 22 | chronic visceral pain[Title/Abstract] | chronic visceral pain:ti,ab | chronic visceral pain:ti,ab | chronic visceral pain:ti,ab | chronic visceral pain:ti,ab | chronic visceral pain:ti,ab |
| 23 | chronic chest pain[Title/Abstract] | chronic chest pain:ti,ab | chronic chest pain:ti,ab | chronic chest pain:ti,ab | chronic chest pain:ti,ab | chronic chest pain:ti,ab |
| 24 | chronic pelvic pain[Title/Abstract] | chronic pelvic pain:ti,ab | chronic pelvic pain:ti,ab | chronic pelvic pain:ti,ab | chronic pelvic pain:ti,ab | chronic pelvic pain:ti,ab |
| 25 | chronic abdominal pain[Title/Abstract] | chronic abdominal pain:ti,ab | chronic abdominal pain:ti,ab | chronic abdominal pain:ti,ab | chronic abdominal pain:ti,ab | chronic abdominal pain:ti,ab |
| 26 | chronic musculoskeletal pain[Title/Abstract] | chronic musculoskeletal pain:ti,ab | chronic musculoskeletal pain:ti,ab | chronic musculoskeletal pain:ti,ab | chronic musculoskeletal pain:ti,ab | chronic musculoskeletal pain:ti,ab |
| 27 | chronic low back pain[Title/Abstract] | chronic low back pain:ti,ab | chronic low back pain:ti,ab | chronic low back pain:ti,ab | chronic low back pain:ti,ab | chronic low back pain:ti,ab |
| 28 | chronic lower back pain[Title/Abstract] | chronic lower back pain:ti,ab | chronic lower back pain:ti,ab | chronic lower back pain:ti,ab | chronic lower back pain:ti,ab | chronic lower back pain:ti,ab |
| 29 | chronic headache[Title/Abstract] | chronic headache:ti,ab | chronic headache:ti,ab | chronic headache:ti,ab | chronic headache:ti,ab | chronic headache:ti,ab |
| 30 | chronic head ache[Title/Abstract] | chronic head ache:ti,ab | chronic head ache:ti,ab | chronic head ache:ti,ab | chronic head ache:ti,ab | chronic head ache:ti,ab |
| 31 | chronic pain[MeSH Terms] | chronic pain MeSH descriptor | chronic pain MeSH descriptor | chronic pain:ti,ab | chronic pain:ti,ab | chronic pain:ti,ab |
| 32 | chronic somatoform pain[Title/Abstract] | chronic somatoform pain:ti,ab | chronic somatoform pain:ti,ab | chronic somatoform pain:ti,ab | chronic somatoform pain:ti,ab | chronic somatoform pain:ti,ab |
| 33 | chronic pelvic girdle pain[Title/Abstract] | chronic pelvic girdle pain:ti,ab | chronic pelvic girdle pain:ti,ab | chronic pelvic girdle pain:ti,ab | chronic pelvic girdle pain:ti,ab | chronic pelvic girdle pain:ti,ab |
| 34 | chronic shoulder pain[Title/Abstract] | chronic shoulder pain:ti,ab | chronic shoulder pain:ti,ab | chronic shoulder pain:ti,ab | chronic shoulder pain:ti,ab | chronic shoulder pain:ti,ab |
| 35 | chronic cervical pain[Title/Abstract] | chronic cervical pain:ti,ab | chronic cervical pain:ti,ab | chronic cervical pain:ti,ab | chronic cervical pain:ti,ab | chronic cervical pain:ti,ab |
| 36 | chronic neck pain[Title/Abstract] | chronic neck pain:ti,ab | chronic neck pain:ti,ab | chronic neck pain:ti,ab | chronic neck pain:ti,ab | chronic neck pain:ti,ab |
| 37 | chronic craniofacial pain[Title/Abstract] | chronic craniofacial pain:ti,ab | chronic craniofacial pain:ti,ab | chronic craniofacial pain:ti,ab | chronic craniofacial pain:ti,ab | chronic craniofacial pain:ti,ab |
| 38 | chronic facial pain[Title/Abstract] | chronic facial pain:ti,ab | chronic facial pain:ti,ab | chronic facial pain:ti,ab | chronic facial pain:ti,ab | chronic facial pain:ti,ab |
| 39 | chronic tension type headache[Title/Abstract] | chronic tension type headache:ti,ab | chronic tension type headache:ti,ab | chronic tension type headache:ti,ab | chronic tension type headache:ti,ab | chronic tension type headache:ti,ab |
| 40 | chronic migraine[Title/Abstract] | chronic migraine:ti,ab | chronic migraine:ti,ab | chronic migraine:ti,ab | chronic migraine:ti,ab | chronic migraine:ti,ab |
| 41 | chronic muscular rheumatism[Title/Abstract] | chronic muscular rheumatism:ti,ab | chronic muscular rheumatism:ti,ab | chronic muscular rheumatism:ti,ab | chronic muscular rheumatism:ti,ab | chronic muscular rheumatism:ti,ab |
| 42 | chronic intractable pain[Title/Abstract] | chronic intractable pain:ti,ab | chronic intractable pain:ti,ab | chronic intractable pain:ti,ab | chronic intractable pain:ti,ab | chronic intractable pain:ti,ab |
| 43 | chronic persistent pain[Title/Abstract] | chronic persistent pain:ti,ab | chronic persistent pain:ti,ab | chronic persistent pain:ti,ab | chronic persistent pain:ti,ab | chronic persistent pain:ti,ab |
| 44 | chronic back pain[Title/Abstract] | chronic back pain:ti,ab | chronic back pain:ti,ab | chronic back pain:ti,ab | chronic back pain:ti,ab | chronic back pain:ti,ab |
| 45 | chronic backache[Title/Abstract] | chronic backache:ti,ab | chronic backache:ti,ab | chronic backache:ti,ab | chronic backache:ti,ab | chronic backache:ti,ab |
| 46 | chronic back ache [Title/Abstract] | chronic back ache:ti,ab | chronic back ache :ti,ab | chronic back ache :ti,ab | chronic back ache :ti,ab | chronic back ache :ti,ab |
| 47 | #1 OR #2 OR #3 OR #4 OR #5 OR #6 OR #7 OR #8 OR #9 OR #10 OR #11 OR #12 OR #13 OR #14 OR #15 OR #16 OR #17 OR #18 OR #19 OR #20 OR #21 OR #22 OR #23 OR #24 OR #25 OR #26 OR #27 OR #28 OR #29 OR #30 OR #31 OR #32 OR #33 OR #34 OR #35 OR #36 OR #37 OR #38 OR #39 OR #40 OR #41 OR #42 OR #43 OR #44 OR #45 OR #46 | #1 OR #2 OR #3 OR #4 OR #5 OR #6 OR #7 OR #8 OR #9 OR #10 OR #11 OR #12 OR #13 OR #14 OR #15 OR #16 OR #17 OR #18 OR #19 OR #20 OR #21 OR #22 OR #23 OR #24 OR #25 OR #26 OR #27 OR #28 OR #29 OR #30 OR #31 OR #32 OR #33 OR #34 OR #35 OR #36 OR #37 OR #38 OR #39 OR #40 OR #41 OR #42 OR #43 OR #44 OR #45 OR #46 | #1 OR #2 OR #3 OR #4 OR #5 OR #6 OR #7 OR #8 OR #9 OR #10 OR #11 OR #12 OR #13 OR #14 OR #15 OR #16 OR #17 OR #18 OR #19 OR #20 OR #21 OR #22 OR #23 OR #24 OR #25 OR #26 OR #27 OR #28 OR #29 OR #30 OR #31 OR #32 OR #33 OR #34 OR #35 OR #36 OR #37 OR #38 OR #39 OR #40 OR #41 OR #42 OR #43 OR #44 OR #45 OR #46 | #1 OR #2 OR #3 OR #4 OR #5 OR #6 OR #7 OR #8 OR #9 OR #10 OR #11 OR #12 OR #13 OR #14 OR #15 OR #16 OR #17 OR #18 OR #19 OR #20 OR #21 OR #22 OR #23 OR #24 OR #25 OR #26 OR #27 OR #28 OR #29 OR #30 OR #31 OR #32 OR #33 OR #34 OR #35 OR #36 OR #37 OR #38 OR #39 OR #40 OR #41 OR #42 OR #43 OR #44 OR #45 OR #46 | #1 OR #2 OR #3 OR #4 OR #5 OR #6 OR #7 OR #8 OR #9 OR #10 OR #11 OR #12 OR #13 OR #14 OR #15 OR #16 OR #17 OR #18 OR #19 OR #20 OR #21 OR #22 OR #23 OR #24 OR #25 OR #26 OR #27 OR #28 OR #29 OR #30 OR #31 OR #32 OR #33 OR #34 OR #35 OR #36 OR #37 OR #38 OR #39 OR #40 OR #41 OR #42 OR #43 OR #44 OR #45 OR #46 | #1 OR #2 OR #3 OR #4 OR #5 OR #6 OR #7 OR #8 OR #9 OR #10 OR #11 OR #12 OR #13 OR #14 OR #15 OR #16 OR #17 OR #18 OR #19 OR #20 OR #21 OR #22 OR #23 OR #24 OR #25 OR #26 OR #27 OR #28 OR #29 OR #30 OR #31 OR #32 OR #33 OR #34 OR #35 OR #36 OR #37 OR #38 OR #39 OR #40 OR #41 OR #42 OR #43 OR #44 OR #45 OR #46 |
| 48 | Hypertension[Mesh] | Hypertension[Mesh] | Hypertension[Mesh] | Hypertension[Mesh] | Hypertension[Mesh] | Hypertension[Mesh] |
| 49 | Blood Pressure[Mesh] | Blood Pressure[Mesh] | Blood Pressure[Mesh] | Blood Pressure[Mesh] | Blood Pressure[Mesh] | Blood Pressure[Mesh] |
| 50 | hypertens*[Title/Abstract] | hypertens*:ti,ab | hypertens*:ti,ab | hypertens*:ti,ab | Hypertens*:ti,ab | Hypertens*:ti,ab |
| 51 | blood pressure[Title/Abstract] | blood pressure:ti,ab | blood pressure:ti,ab | blood pressure:ti,ab | blood pressure:ti,ab | blood pressure:ti,ab |
| 52 | #48 OR #49 OR #50 OR #51 | #48 OR #49 OR #50 OR #51 | #48 OR #49 OR #50 OR #51 | #48 OR #49 OR #50 OR #51 | #48 OR #49 OR #50 OR #51 | #48 OR #49 OR #50 OR #51 |
| 55 | #47 AND #52 | #47 AND #52 | #47 AND #52 | #47 AND #52 | #47 AND #52 | #47 AND #52 |

# **Table S2. Study Characteristics**

| **Author & Year** | **Sample population** | **Study type** | **Country** | **Sample size; n included** | **Average age** (mean; SD) | F**emale** (%) |
| --- | --- | --- | --- | --- | --- | --- |
| Adamo 2023 | 242 patients with BMS presenting to a dental clinic. 242 age- and sex-matched controls. | Retrospective case-control | Italy | 500; 484 | CP: 65.61; 12.7  Control: 64.34; 10.4  p=0.231* | 50 |
| Atzeni 2023 | 62 female outpatients with FMS. 4,093 age-matched female controls from a representative Italian population sample. | Cross-sectional | Italy | 4,155; 4,155 | CP: 53.7; 10.7  Control: NR | 100 |
| Canfora 2022 | 250 female patients with BMS recruited by an oral medicine department. 250 age-matched female controls. | Retrospective case-control | Italy | 535; 500 | CP: 62.3; 11.4  Control: 60.8; 11.7  p=0.148* | 100 |
| Chang 2015 | 25,969 patients with FMS and no psychiatric history and 103,876 age- and sex-matched controls (from the Taiwan National Health Insurance Research Database). Study 1 data used. | Prospective matched cohort | Taiwan | 129,845; 129,845 | 46.87; 15.92 | 59.9 |
| Chen 2012 | 948 patients with chronic migraine and 3790 age-, sex- and income- matched controls (from Taiwan National Health Insurance Research Database). | Retrospective matched cohort | Taiwan | 4,891; 4,738 | NR; NR  Grouped: 7.3%<=20y, 40.3% 20-40y, 37.6% 40-60y, 14.8% >=60y).  Range 20-59 years. | 80 |
| Chen 2014 | 752 female patients with BPS/IC and 3,760 age-matched female controls (from the Taiwan Longitudinal Health Insurance Database 2000). | Prospective cohort | Taiwan | 1,000,000; 4512 | 47.3; 15.4 | 100 |
| Chudek 2024 | Elderly Polish population sample (from national multicentre study on ageing). | Retrospective cohort | Poland | 4,979; 3473 | 78.5; 8 | 47.5 |
| Cristofolini 2008 | Patients with end-stage renal disease receiving haemodialysis 3 times per week (recruited from dialysis unit). | Cross-sectional | Brazil | 234; 205 | CP: 54.5; 13.8  Control: 49.4; 15.8  p=0.02* | 49.3 |
| Foley 2021 | Canadian residents eligible for Medical Care Plan benefits (health administrative data). | Retrospective cohort | Canada | 516,729; 504,693 | CP: 50.8; 18.1  Control: 37.5; 21.1  p<0.001* | 50.9 |
| Goodson 2013 | Volunteer-based family-structured population sample (Generation Scotland: The Scottish Family Health study). | Cross-sectional | UK | 23,960; 13,328 | 48; NR  Range 36-59 | 58.6 |
| Ha 2014 | Nationally representative population sample of south Koreans aged 20-89. | Cross-sectional | South Korea | 13,841; 13,841 | Male: 47.8; 15.4  Female: 48.3; 16 | 56.1** |
| Hagen 2005 | General population sample (2 consecutive public health studies: HUNT-1 & HUNT-2). HUNT-2 crude data of SBP ≥140 used for this analysis. | Prospective epidemiologic cohort | Norway | 47,556; 46,901 | NR; NR  Range 20-80 | 53.3** |
| Heuch 2014 | General population sample (from health survey, HUNT-2 cross-sectional and HUNT-3 prospective). Cross-sectional data from HUNT-2 used for this analysis. | Cross-sectional & prospective | Norway | 39,872 (cross-sectional) | NR; NR  Range 30-69 | 55.2** |
| Kakihana 2021 | General population sample (circulatory risk study). | Cross-sectional | Japan | 2,970; 2,845 | NR; NR  Grouped: 12.3% 40-49, 13.3% 50-59, 34.5% 60-69, 8.4% 80-89 | 62 |
| Kim 2021 | 58 female FMS patients (from rheumatology outpatient clinic). 158 healthy controls (from annual health check-up service). | Retrospective cross-sectional | Italy | 216; 216 | CP: 53.2; 12.2  Control: 57.4; 9.7 | 100 |
| Meert 2024 | General population sample (from longitudinal cohort study). | Longitudinal cohort | Netherlands | 4,519; 3,437 | 55.11; 9.87 | 52.7 |
| Mohammadi 2021 | General population sample (part of national prospective epidemiological study). | Cross-sectional | Iran | 10,000; 9,932 | 49.9; 9.56 | 53.5 |
| Morales-Epinoza 2016 | 130 patients with CWP and 124 age- and sex-matched controls. Selected from patients attending 3 urban primary care centres in Spain. | Retrospective case-control | Spain | 16,229; 154 | CP: 61.7; 11.7  Control: 60.5; 11.3 (control) | 94.1** |
| Parlatescu 2023 | 99 patients with BMS and 88 controls aged ≥50 years recruited from Dentistry faculty in Romania. | Retrospective case-control | Romania | 187; 187 | 64.03; 7.833  Range 50-85 | 82.9 |
| Tsai 2019 | 17.568 patients ≥65 years with chronic pain and 17568 age and sex-matched controls (Longitudinal Health Insurance Database 2000, a subset of the Taiwan National Health Insurance Research Database). | Retrospective matched cohort | Taiwan | 2,000,000; 35136 | 73.5; 5.7 | 55.1 |
| Tseng 2016 | 47,279 patients with newly diagnosed FMS between 2000 and 2002 and 189,112 age- and sex-matched controls (Longitudinal Health Insurance Database 2000, a subset of the Taiwan National Health Insurance Research Database). | Retrospective matched cohort | Taiwan | 1,000,000; 236391 | CP: 44.7; 16.3  Control: 44.6; 16.4 | 59.5 |
| Tsepilov 2023 | 21,543 patients with CBP and 98,647 controls (from UK Biobank). | Retrospective case-control | UK | 120,217; 120,217 | NR; NR  Range 40-69 | NR |
| Wang 2024 | Two-sample Mendelian Randomisation study using GWAS data from UK BioBank. | Retrospective case-control | UK | 1,873,960 | NR; NR | NR |

BMS, burning mouth syndrome; FMS, fibromyalgia syndrome; BPS/IC, bladder pain syndrome/interstitial cystitis; CP, chronic pain; CWP, chronic widespread pain; CBP, chronic back pain; SD, standard deviation; NR, not recorded. *P-value for difference as taken from paper. **As calculated from study data.

# **Table S3: Risk of bias assessment (ROBINS-E)**

| **First Author & Year** | **Domain 1: Confounding** | **Domain 2: Exposure Measurement** | **Domain 3:**  **Participant Selection** | **Domain 4:**  **Post-exposure interventions** | **Domain 5:**  **Missing Data** | **Domain 6:**  **Outcome Measurement** | **Domain 7:**  **Selection of Reported Result** | **Overall Risk of Bias** |
| --- | --- | --- | --- | --- | --- | --- | --- | --- |
| **Adamo 2023** | Low | Low | Some concerns | Low | Low | Low | Low | **Some concerns** |
| **Atzeni 2023** | Low | Low | Low | Low | Low | Low | Low | **Low** |
| **Canfora 2022** | Low | Low | Some concerns | Low | Low | Low | Low | **Some concerns** |
| **Chang 2015** | Low | Low | Some concerns | Some concerns | Low | Low | Low | **Some concerns** |
| **Chen 2012** | Low | Low | Low | Low | Low | Low | Low | **Low** |
| **Chen 2014** | Low | Low | Some concerns | Low | Low | Low | Low | **Some concerns** |
| **Chudek 2024** | High | Some concerns | Some concerns | Low | Low | Low | Low | **High** |
| **Cristofolini 2008** | High | Some concerns | High | Low | Low | Low | Low | **High** |
| **Foley 2021** | Some concerns | Some concerns | Some concerns | Low | Low | Low | Low | **Some concerns** |
| **Goodson 2013** | Low | Some concerns | Low | Low | Low | Low | Low | **Some concerns** |
| **Ha 2014** | Low | Some concerns | Low | Low | Low | Low | Low | **Some concerns** |
| **Hagen 2005** | High | Some concerns | Low | Low | Low | Low | Low | **High** |
| **Heuch 2014** | High | Some concerns | Low | Low | Low | Low | Low | **High** |
| **Kakihana 2021** | Low | Some concerns | Some concerns | Low | Low | Low | Low | **Some concerns** |
| **Kim 2021** | High | Low | Some concerns | Low | Low | Low | Low | **High** |
| **Meert 2024** | Low | Some concerns | Low | Low | Low | Low | Low | **Some concerns** |
| **Mohammadi 2021** | Low | Some concerns | Low | Low | Low | Low | Low | **Some concerns** |
| **Morales-Epinoza 2016** | Low | Some concerns | Some concerns | Low | Some concerns | Low | Low | **Some concerns** |
| **Parlatescu 2023** | High | Some concerns | Some concerns | Low | Low | Low | Low | **High** |
| **Tsai 2019** | Low | Some concerns | Low | Low | Low | Low | Low | **Some concerns** |
| **Tseng 2016** | Low | Low | Low | Low | Low | Low | Low | **Low** |
| **Tsepilov 2023** | Low | Low | Low | Low | Low | Low | Low | **Low** |
| **Wang 2024** | Some concerns | Low | Low | Low | Low | Low | Low | **Some concerns** |

# **Table S4. Study details of exposure and outcome and associated odds ratios**

| **Author & Year** | **CP type** | **CP definition** | **HTN Definition** | **Exposed**  **(CP)**  ***n*** | **Controls**  **(non-CP)**  ***n*** | **Exposed**  **with HTN**  ***n*** | **Controls with HTN**  ***n*** | **OR** |
| --- | --- | --- | --- | --- | --- | --- | --- | --- |
| **Adamo 2023** | Burning Mouth Syndrome | International Classification of Orofacial Pain (ICOP 2020) 1st edition | SBP ≥140mmHg, DBP ≥90 mmHg or taking antihypertensives | 242 | 242 | 133 | 81 | 2.43  (unadjusted) |
| **Atzeni 2023** | Fibromyalgia syndrome | Modified 2010 American College of Rheumatology criteria for FMS | SBP ≥140mmHg or DBP ≥90 mmHg (mean of two consecutive blood pressure values) or taking antihypertensives | 62 | 4,093 | 32 | 1,547 | 1.76  (unadjusted) |
| **Canfora 2022** | Burning Mouth Syndrome | International Classification of Orofacial Pain (ICOP 2020) 1st edition | SBP ≥140mmHg or DBP≥90 mmHg (mean of two consecutive blood pressure values taken by a physician) or taking antihypertensives | 250 | 250 | 128 | 76 | 2.4  (unadjusted) |
| **Chang 2015** | Fibromyalgia syndrome | ICD-9-CM code 729.1, diagnosed by neurologists, rheumatologists, rehabilitation doctors, or pain specialists | ICD-9-CM diagnostic code | 25,969 | 103,876 | 8,424 | 2,5770 | 1.46  (unadjusted) |
| **Chen 2012** | Chronic migraine | Patients with at least one neurological outpatient visit with primary or secondary 2007 ICD-9-CM code 346.11 and who were diagnosed by a certified neurologist at a medical centre between 2007 and 2008 | Diagnosis as per ICD-9-CM codes 401.0, 401.1, 401.9, 402-405, 437.2 | 948 | 3,790 | 173 | 462 | 1.61*  (unadjusted) |
| **Chen 2014** | Bladder pain syndrome/interstitial cystitis | ICD-9-CM code 595.1 (chronic interstitial cystitis) | ICD-9-CM diagnostic code | 752 | 3,760 | 186 | 683 | 1.48 (unadjusted) |
| **Chudek 2024** | Chronic widespread pain and chronic regional pain | Self-reported pain that lasted more than 3 months. CWP if pain experienced in axial region and any part of both lower and upper limbs. CRegP if criteria for CWP were not met. | As per 2013 European Society of Cardiology Guidelines for Management of Arterial Hypertension. Average of 4 measurements performed on 2 separate visits, or the use of antihypertensives. | 1,483 (306 CWP, 1177 CRegP) | 1,990 | 1,115 | 1,458 | 1.11 (unadjusted)  CWP: 1.29 (0.97-1.72), CRegP: 1.06 (0.9-1.25) |
| **Cristofolini 2008** | Chronic lower back pain | Current complaints of lumbar pain in the dorsal region between the last back arch and the gluteal crease, not related to infections, tumours or fractures, and lasting more than 3 consecutive months | From medical records and interviews with patients and the average of three measurements of pre-dialysis blood pressure ≥140/90 mm Hg or normal blood pressure with the use of antihypertensives | 74 | 131 | 65 | 96 | 2.63 (unadjusted) |
| **Foley 2021** | Various – musculoskeletal/arthritis, back/neck, headache, musculoskeletal trauma, neuropathic, bone, other | The Chronic Pain Algorithm: a single claim date with an anaesthetist recording a chronic pain–related provincial Medical Care Plan procedure billing code, or 5 or more claim dates with any physician recording any pain-related diagnostic code in a 5-year period with more than 183 days separating at least 2 pain-related claim dates | Canadian Chronic Disease Surveillance System administrative data case definition: two or more physician claims within two years, or one inpatient hospital admission listing hypertension as a diagnosis, both with the relevant ICD code for hypertension | 184,580 | 320,113 | 70,602 | 49,297 | 3.25 (adjusted: sex, regional health authority, rural/urban residential location) |
| **Goodson 2013** | Various – head, neck, back, chest, abdomen, limbs, and other sites  of pain not specified | Self-reported pain or discomfort lasting >3 months | SBP ≥140mmHg or DBP ≥90mmHg, measured on 2 occasions sitting for at least 10mins | 5,209 | 8,119 | NR | NR | SBP: 0.99  DBP: 1.15  (both adjusted by age and gender) |
| **Ha 2014** | Chronic lower back pain | Self-reported episodes of back pain lasting three months or longer during the previous year | SBP >140 mmHg, DBP >90 mmHg, or taking antihypertensives | 2,294 | 11,547 | 788 | 2,928 | 1.54*  (unadjusted) |
| **Hagen 2005** | Chronic musculoskeletal pain  (Using HUNT-2 data for SBP ≥ 140 mmHg) | Self-reported - “Have you during the last year continuously for at least 3 months had pain and/or stiffness in muscles and joints?” | Divided into categories: 140-159 and ≥160 for SBP, and 90-99 and ≥100 for DBP. | 23,998 | 22,662 | 11,233 | 10,577 | 1.01*  (unadjusted) |
| **Heuch 2014** | Chronic lower back pain | Self-reported lower back pain persisting for at least 3 months continuously during the last year | Grouped - >=160 SBP, 140-159 SBP, >-100 DBP, 90-99 DBP | 10,028 | 29,844 | 2,953  (SBP)  1,522  (DBP) | 8,998  (SBP)  10,429 (DBP) | SBP ≥140mmHg:  0.97 (unadjusted)  DBP ≥90mmHg:  0.33 (unadjusted) |
| **Kakihana 2021** | Chronic lower back pain (CLBP) and chronic knee pain (CKP) | Self-reported pain occurring in the past 4 weeks and lasting >3 months | SBP ≥140mmHg and/or DBP ≥90mmHg and/or on antihypertensives | 919  (CKP)  1,114 (CLBP) | 1,926  (CKP)  1,728 (CLBP) | 519  (CKP)  591  (CLBP) | NR | CKP: 1.08  CLBP: 0.93  (adjusted: age, sex, area, overweight, inactivity, smoking status, drinking status, mental stress, depressive status, job) |
| **Kim 2021** | Fibromyalgia syndrome | Revised American College of Rheumatology criteria | SBP ≥140mmHg, DBP ≥90mmHg, or taking antihypertensives. Two independent physicians gathered data on hypertension, diabetes, and dyslipidemia by reviewing the electronic medical records. | 58 | 158 | 41 | 112 | 0.99  (unadjusted) |
| **Meert 2024** | Chronic musculoskeletal pain | Self-reported - "Have you experienced pain in any of these regions for a duration exceeding three months in the last 12 months?" | Systolic ≥140 mmHg and/or diastolic ≥90 mmHg and/ or taking antihypertensives | 350 | 1,372 | NR | NR | 0.94  (adjusted: sex and age) |
| **Mohammadi 2021** | Chronic primary headache | Self-reported – Headaches that occur 15 days a month for >=3 months. | SBP >=140mmHg or DBP >=90mmHg or previous diagnosis of hypertension +/- treatment with antihypertensives | 741 | 9,191 | 237 | 1,998 | 1.52  (adjusted: age, gender, education, occupation, smoking, alcohol, opioids, BMI, physical activity, cholesterol, diabetes mellitus, triglycerides, FHx chronic headache) |
| **Morales-Epinoza 2016** | Chronic widespread pain | American College of Rheumatology criteria for CWP - pain must have been present for at least 3 months and be present in 2 contralateral areas of the body, above and below the waist and in the axial skeleton | SBP >140 mm Hg or DBP >90 mm Hg, from medical records. | 130 | 124 | 58 | 39 | 1.76  (unadjusted) |
| **Parlatescu 2023** | Burning Mouth Syndrome | The daily presence of at least one of the oral symptoms (burning sensation, pain, and itching sensation) for more than 2h for at least 3 months | SBP ≥140mmHg and/or DBP ≥90mmHg following repeated examinations | 99 | 88 | 84 | 16 | 25.2  (unadjusted) |
| **Tsai 2019** | Various – osteoarthritis (24.2%), spinal disorders (22.4%), peripheral vascular disease (14%), osteoporosis (9.5%), gout, malignancy, headache, diabetic neuropathy, rheumatoid arthritis, pressure ulcer.  Classified according to ICD-9-CM codes. | Patients who have used common analgesics (acetaminophen, NSAIDs excluding aspirin, or opioids) for at least 3 months | SBP ≥ 140 mmHg or DBP ≥ 90 mmHg or taking antihypertensives | 17,568 | 17,568 | 9,839 | 6,667 | 2.08  (unadjusted) |
| **Tseng 2016** | Fibromyalgia syndrome | ICD-9-CM codes 729.0 and 729.1 | ICD-9-CM code as per NHIRD | 47,279 | 189,112 | 10,550 | 31,212 | 1.45  (unadjusted) |
| **Tsepilov 2023** | Chronic back pain | UK Biobank definitions | UK Biobank definition | 21,543 | 98,674 | NR | NR | 1.12  (adjusted: sex, age, genotyping batch) |
| **Wang 2024** | Various - chronic headache, chest pain, abdominal pain, joint pain, back pain, limb pain, multisite chronic pain | UK Biobank definitions | UK Biobank definitions of essential and secondary hypertension. Essential hypertension used for this analysis. | Grouped: 4,293 (chronic headache), 73,061 (chronic chest pain), 21,711 (chronic abdominal pain), 1,451 (chronic joint pain), 80,588 (chronic back pain), 34,007 (chronic limb pain). | Grouped: 458,717 (chronic headache), 384,951 (chronic chest pain), 1,7200 (chronic abdominal pain), 461,559 (chronic joint pain), 36,816 (chronic back pain), 299,606 (chronic limb pain) | NR | NR | Multisite chronic pain: 1.034  Chronic chest pain: 1.22  Chronic headache: 1.52  Abdominal pain: 1.00  Joint pain: 0.60  Back pain: 1.01  Limb pain: 1.00  (unadjusted) |

CP, chronic pain; HTN, hypertension; OR, odds ratio; SBP, systolic blood pressure; DBP, diastolic blood pressure; CWP, chronic widespread pain; CRegP, chronic regional pain; NR, not recorded; ICD-9-CM, international classification of diseases, 9^th^ revision, clinical modification. *Calculated from study data.

# Figure S1: Directed Acyclic Graph (DAG)

Showing the assumptions made for considering confounding variables. Made using ‘DAGiity’ ^2^ according to principles of causal inference ^3^.


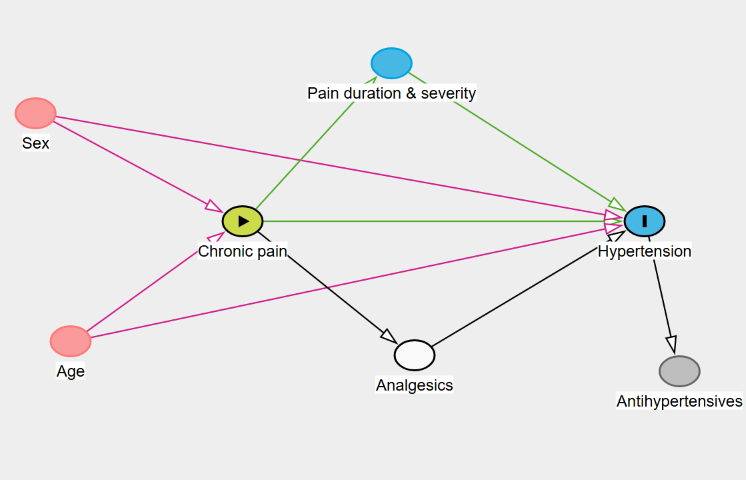


# Figure S2: L’Abbé plot comparing CP and non-CP event rates (HTN) across studies.


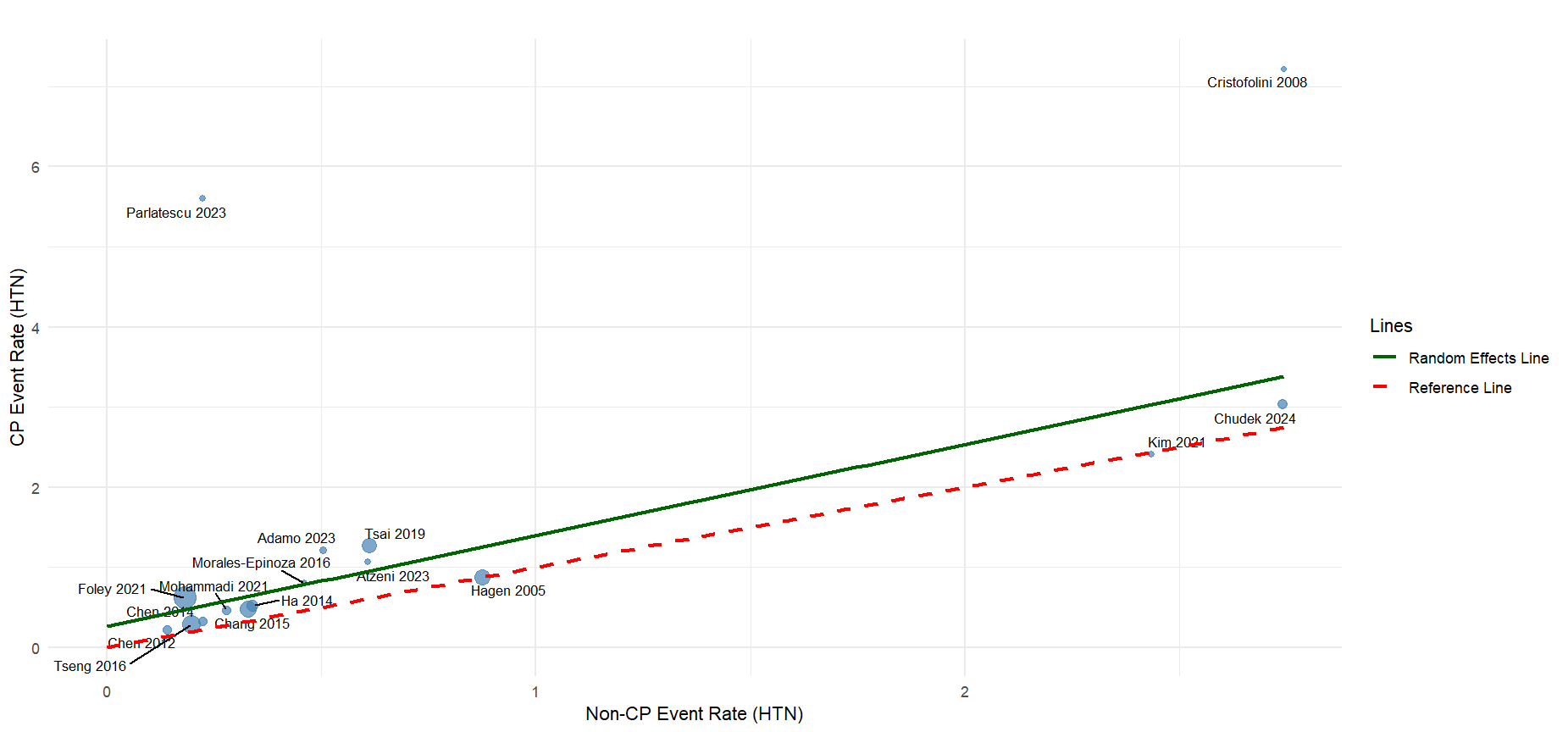


# Figure S3: Funnel plot of study Odds Ratio against the Standard Error


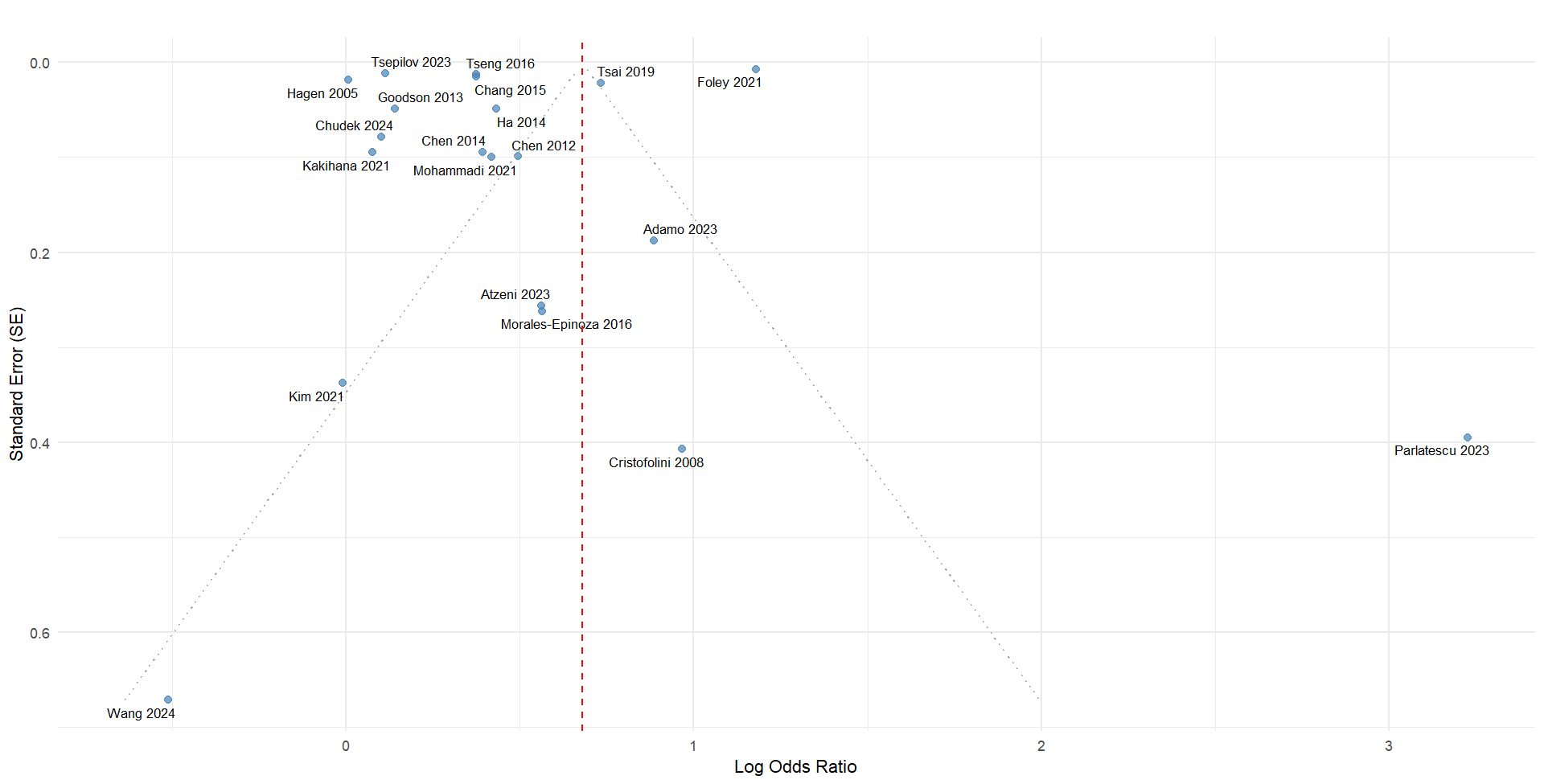


# Figure S4: Conceptual diagram illustrating the pathways involved in HTN-associated hypoalgesia

*
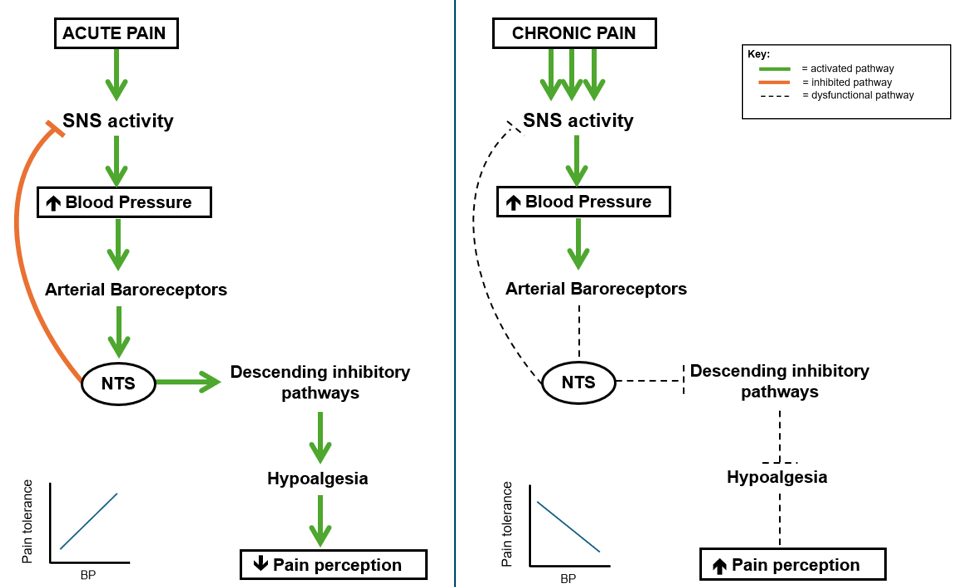
*

# **References**

1 Clark JM, Sanders S, Carter M, et al. Improving the translation of search strategies using the Polyglot Search Translator: a randomized controlled trial. *J Med Libr Assoc* 2020; **108**: 195-207, doi: <10.5195/jmla.2020.834>

2 Textor J, van der Zander B, Gilthorpe MS, Liśkiewicz M, Ellison GT. Robust causal inference using directed acyclic graphs: the R package ‘dagitty’. *Int J Epidemiol* 2017; **45**: 1887-94, doi: <10.1093/ije/dyw341>

3 Stovitz SD, Shrier I. Causal inference for clinicians. *BMJ Evid-Based Med* 2019; **24**: 109-12, doi: <10.1136/bmjebm-2018-111069>
